# A Prosthetic Shank With Adaptable Torsion Stiffness and Foot Alignment

**DOI:** 10.3389/fnbot.2020.00023

**Published:** 2020-05-08

**Authors:** Jochen Schuy, Nadine Stech, Graham Harris, Philipp Beckerle, Saeed Zahedi, Stephan Rinderknecht

**Affiliations:** ^1^Continental Teves AG & Co. oHG, Frankfurt am Main, Germany; ^2^seleon gmbh, Heilbronn, Germany; ^3^Chas a Blatchford & Sons Ltd, Basingstoke, United Kingdom; ^4^Institute for Mechatronic Systems, Mechanical Engineering, Technische Universität Darmstadt, Darmstadt, Germany; ^5^Elastic Lightweight Robotics Group, Robotics Research Institute, Department of Electrical Engineering and Information Technology, Technische Universität Dortmund, Dortmund, Germany

**Keywords:** lower limb prosthesis, bioinspiration, elastic actuation, user-specific interaction, torsion adapter, impedance control

## Abstract

Torsion adapters in lower limb prostheses aim to increase comfort, mobility and health of users by allowing rotation in the transversal plane. A preliminary study with two transtibial amputees indicated correlations between torsional stiffness and foot alignment to increase comfort and stability of the user depending on the gait situation and velocity. This paper presents the design and proof-of-concept of an active, bio-inspired prosthetic shank adapter and a novel approach to create a user-specific human-machine interaction through adapting the device's properties. To provide adequate support, load data and subjective feedback of subjects are recorded and analyzed regarding defined gait situations. The results are merged to an user individual preference-setting matrix to select optimal parameters for each gait situation and velocity. A control strategy is implemented to render the specified desired torsional stiffness and transversal foot alignment values to achieve situation-dependent adaptation based on the input of designed gait detection algorithms. The proposed parallel elastic drive train mimics the functions of bones and muscles in the human shank. It is designed to provide the desired physical human-machine interaction properties along with optimized actuator energy consumption. Following test bench verification, trials with five participants with lower limb amputation at different levels are performed for basic validation. The results suggest improved movement support in turning maneuvers. Subjective user feedback confirmed a noticeable reduction of load at the stump and improved ease of turning.

## 1. Introduction

Humans with lower limb amputations require adequate motion support for societal participation. The relevance of this issue is highlighted by the forecast that 3.6 million people with such amputations will live in the US in 2050 (Ziegler-Graham et al., 2008). In the last two decades, the development of lower limb prostheses focused on supporting users in gait situations with straight direction like straight walking on level ground or climbing stairs and ramps.

Therefore, several micro-processed and actively supporting products are available (Windrich et al., 2016). In contrast, less attention was paid to dynamic gait situations, e.g., turning maneuvers, which add up to 40% of daily gait situations (Glaister C. et al., 2007). Especially in these situations, high load and shear stress on the residual limb of the amputee often result in skin and soft tissue problems. This causes a massive discomfort and reduces amputees' satisfaction with the support of the device.

Current commercial products are passive rotational adapters that reduce torsional load peaks (Twiste and Rithalia, 2003; Flick et al., 2005). In consequence shear stress at the residual limb is decreased and pain might be alleviated (Lamoureux and Radcliffe, 1977; Van der Linden et al., 2002; Gard and Konz, 2003; Segal et al., 2009, 2014). In addition, such adapters facilitate a more physiological gait (Stauf, 2000; Segal et al., 2011, 2014) with increased gait symmetry (Lamoureux and Radcliffe, 1977), reduced step width (Su et al., 2010), and improved roll over (Ross et al., 2003). The additional transversal degree of freedom supports users during turning maneuvers (Gard and Konz, 2003; Segal et al., 2009, 2014), which is shown by increased step length and stability (Segal et al., 2011). Passive rotational adaptaters have been shown to reduce transversal knee and hip torques during ipsilateral circling and to improve stability during contralateral circling (Segal et al., 2009, 2010, 2011). Moreover, such adapters can decrease tissue stress and breakdown risk, increase user mobility, and decrease the incidence of falls (Flick et al., 2005). However, the effects of rotational adapters are not fully, statistically confirmed and appear to be strongly user-specific (Segal et al., 2009, 2010, 2011; Heitzmann et al., 2015). Yet, passive systems lack the adaptive nature of their biological counterparts and recent research considers mimicking this versatility and indicates that additional benefits can be expected from semi-active or active rotational adapters (Gard and Konz, 2003; Glaister et al., 2009; Segal et al., 2009; Glaister, 2012; Orendurff, 2012; Pew, 2014; Olson and Klute, 2015; Pew and Klute, 2015, 2017b; Price et al., 2019).

Research reports (Segal et al., 2009, 2014) show the need of a more detailed determination of optimal support during dynamic gait situations (Schuy, 2016). Up to now, research focused on self-selected walking speeds (Segal et al., 2009, 2010, 2011) and rotation adapters have been shown to impact abrupt movements (Heitzmann et al., 2015). Variable stiffness seems to be necessary to mimic natural gait (Hansen et al., 2004) and can, thus, be expected to improve mobility. Research by Pew and Klute (2015) outlines that passive devices lack dynamic adaptation and Schuy (2016) shows that preferred stiffness values depend on gait velocity, gait situation, and the users themselves. In Olson and Klute (2015), design and bench testing of a series elastic actuator (SEA) for transverse plane control is described. Its motor housing is designed as an elastic element including strain gauges to build up a torque transducer. First bench tests of the system for impedance controlled stiffness adaption yielded promising results. Another semi-active adapter for stiffness adaptation is presented in Pew and Klute (2015, 2017b). Adaptive stiffness is realized by displacement of the pivot of a lever that transmits force to the rotational spring.

In contrast to previous approaches, this paper presents a prosthetic shank adapter that supports the user during turning maneuvers by adaptation of torsional stiffness and transversal foot alignment. This is facilitated by combining bio-inspired elastic actuation for energy efficiency (Van Ham et al., 2009), user-specific control, and interaction behavior. Section 2 describes the development method and process. A novel test cycle based on typical loads in daily living is introduced in Section 3 and applied for modeling and simulation. Sections 4 and 5 focuses on a proof-of-concept evaluation of the control and mechatronic design of the shank adapter, which is experimentally evaluated in trials with five amputees in Section 6. Finally, Section 7 discusses the main conclusions and contributions of the paper.

## 2. Method of Development Process

In this section, the method of the development of the adaptive prosthetic shank adapter is explained. A detailed examination of users' needs and biomechanical lower limb functions during dynamic gait situations is performed to determine optimal support requirements. Therefore, a mobile measurement system was used to capture load and motion in six degrees of freedom within the prosthesic structure (Schuy and Rinderknecht, 2014; Schuy et al., 2014). The development relies on motion and load data acquired from two users with transtibial amputation wearing passive torsion adapters (TT Pro, Chas A. Blatchford & Sons Ltd., UK) in a preliminary study (Schuy, 2016). The participants performed various dynamic gait situations, e.g., cornering and walking circles of different diameters (0.5, 1 m) in both directions (ipsilateral, contralateral) at self-selected velocities (slow, medium, fast). Between the experiments, the configuration of torsional stiffness (soft, middle, stiff) and foot alignment (-6°, neutral, 6°) of the passive torsion adapter was varied. The stiffness shows nonlinear, degressive characteristics, where soft, middle, and hard stiffness settings corresponded to 0.3, 0.5, and 0.6*Nm*/° for the linear part found roughly at 0 − 5°. Load and motion data are analyzed and juxtaposed to the subjective feedback of the participants. Thereby, the configuration of the adapter, gait situation, and gait velocity can be related to the load in the prosthetic structure as well as to the subjective stability and comfort of the users. Based on these insights, transversal-plane support during dynamic gait situation is specified in terms of stiffness preferences during stance phase and foot alignment during swing (Schuy, 2016). The user-specific optimal configuration is determined for each considered gait situation and velocity, which leads to an individual preference-setting matrix (IPSM) for parameter adaptation. An excerpt of an IPSM is presented in Table 1, favoring an external foot alignment and soft stiffness setting for an ipsilateral 90° turn at medium velocity. For the same turn in contralateral direction, a medium stiffness setting and neutral foot alignment is preferred. Consequently, the IPSM stores user-specific preferences, which can be selected after detection of gait situation and velocity to consider user preference.

**Table 1:** Excerpt of an exemplary individual preference-setting matrix (Stuhlenmiller et al., 2017).

**Situation**	**Velocity**	**Stiffness**	**Foot alignment**
	Slow	Medium	External
90° turn, Ipsilateral	Medium	Soft	External
	Fast	Soft	External
	Slow	Medium	Neutral
90° turn, Contralateral	Medium	Medium	Neutral
	Fast	Medium	Neutral

*Each combination of gait situation and velocity is conjugated with the corresponding user-specific optimal torsional stiffness and foot alignment*.

**Figure 1:**
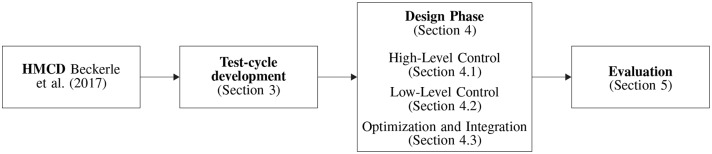
Overview of development, realization, and testing of the prosthetic shank adapter as presented in this paper.

In order to realize the preferred stiffness and foot alignment, a prosthetic shank adapter is developed according to the process depicted in Figure 1. Using the human-machine-centered design method (HMCD) (Beckerle et al., 2017), development priorities are determined while considering human factors. According to the prioritization elaborated in Schuy (2016), actuation and gait detection are focused in the subsequent design of the prosthetic shank adapter. To take different gait situations and velocities into account, a test cycle that models a typical day of an amputee is generated. This test cycle facilitates the comparison of actuator designs regarding their efficiency, supports control design, and is used in bench testing. The development of the adaptive prosthetic shank adapter is split into several phases, focusing on high-level control, low-level control, and mechatronic design. For validation of hardware and control strategy, test-bench trials are performed.

## 3. Test-Cycle Development for Design, Dimensioning, and Testing

A novel approach is presented in this section to generate test-cycles as a tool to develop and evaluate lower limb prostheses. The proposed approach is based on estimated everyday loads that are common for users with lower limb amputation. Instead of utilizing averaged data for one step, movement protocols are generated from distributions of daily gait situations. Thereby, more realistic loads can be taken into account in the design of lower limb prostheses due to consideration of data for initializing and end of movements. This method is also related to test-cycles used by the automotive industry, e.g., the Federal Test Procedure (Office of Mobile Sources and Offif of Air & Radiation, 1993).

Existing studies of amputees' behavior in daily life have been analyzed to obtain an overview of an amputee's common day (Sedgman et al., 1994; Nietert et al., 1997, 1998; Lelas et al., 2003; Taylor et al., 2005; Klute et al., 2006; Taylor, 2006; Glaister B. et al., 2007; Glaister C. et al., 2007; Glaister et al., 2009; Stepien et al., 2007; Oehler, 2015; Strike and Taylor, 2009; Segal et al., 2011; Halsne et al., 2013; Hordacre et al., 2014). The average number of steps is assumed to be 6,500 for transtibial amputees according to Tudor-Locke and Myers (2001), Stepien et al. (2007), Halsne et al. (2013), and Segal et al. (2014). Considering the gait situation, every single step can be assigned to a task with translational or rotational movement as well as static situations. Possible categorizations of those tasks are analyzed according to the daily number of steps and situation distribution of amputees described in Glaister C. et al. (2007), Oehler (2015), and Hordacre et al. (2014). The percentage of translational gait situations is assumed to be 60 % of all movements. It can be divided in 55 % straight gait and 5 % managing height differences (Glaister C. et al., 2007).

To the best of the authors' knowledge, daily distributions of different turning activities are not known for amputees, thus, distributions of able-bodied persons are assumed for the test cycle. According to Glaister C. et al. (2007), 40 % of movements in daily activities are turnings, which can be further distinguished into 45°, 90°, 180°, and 360° turns (Sedgman et al., 1994). The average radius of turns during daily activities is approximately 1 m (Lelas et al., 2003; Segal et al., 2014).

In addition to statistic distribution of straight steps and turns, a typical day can be down-scaled to the environment classes *Home, Office, Shop*, and *Cafeteria* (Schuy, 2016). *Transition* classes are included to consider changes between environment classes. Each environment class consists of movement protocols, containing type and order of gait situations, e.g., three steps of straight walking followed by a 90° turn. By sequencing motion, velocity, and load of single gait situations, measured in preliminary experiments (Schuy, 2016), a representation of the usage during an average day is generated.

In Table 2, steps and gait situations of typical movement protocols are summarized for different environment classes (Schuy, 2016). Furthermore, the total number of turns for each environment class is split into each turn angle category according to the percentage listed in Table 3.

**Table 2:** Relative frequency and number of daily steps classified by the environments employed to generate the test cycle, representing an average day of an amputee.

	**Home**	**Office**	**Shop**	**Cafeteria**
Relative frequency	68 %	16 %	11 %	5 %
Number of steps	4, 420	1, 040	715	325
Straight	60 %	55 %	65 %	50 %
Turns	40 %	45 %	35 %	50 %

**Table 3:** Relative frequency and number of steps of gait situations during an average day of an amputee, which is used as the basis for the proposed test-cycle.

	**Straight**		**Turning**			
Situation	Level walking	Stairs	45°	90°	180°	360°
Relative frequency	55 %	5 %	2.4 %	28.8 %	8.8 %	< 1 %
Number of steps	3,575	325	156	1,872	572	–

Aggregating gait data of single maneuvers according to the movement protocols for each environment and satisfying the presented statistical distributions provides a test-cycle representing typical motions for one day. This procedure supports the automatic generation of test-cycles to design an assisting device with respect to the whole day instead of focusing on specific gait situation. In addition, bench-testing of control and hardware for a holistic evaluation of the device is facilitated. Specifically for this work, the test-cycles allow a proof of concept development of user-specific adaptation of the prosthetic shank adapter to different gait situations.

## 4. Development and Realization of a Prosthetic Shank Adapter

To assist users during turning maneuvers, a prosthetic shank adapter with adaptive torsional stiffness and foot alignment is suggested. The design of this device is performed with a test-cycle aggregated from data given in Table 3 and thereby considers the relevant share of turning in everyday living. The required function is to adapt torsional stiffness during stance phase and set foot alignment during swing phase. In both cases, the desired values are determined from the users' preferences and depend on gait situation and velocity. Based on the preliminary study (Schuy, 2016), the torsional stiffness should be dynamically adjustable from 0.2 N m/° to 1.8 N m/° with maximum foot alignment of ±6°.

**Figure 2:**
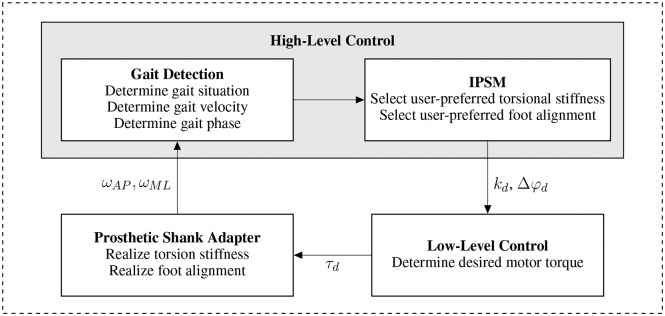
System overview for the prosthetic shank adapter. Angular shank velocity in sagittal and frontal plane are measured for the gait detection algorithm and fed into the high-level control scheme. Based on the detected gait situation, velocity, and phase, the user-preferred torsional stiffness and foot alignment are selected and forwarded to the low-level controller. The low-level control algorithm and actuator realize the user-specific system behavior.

Figure 2 gives an overview of the realized hardware system and the corresponding low-level and high-level control algorithms. The high-level control contains a gait detection algorithm evaluating signals from an inertial measurement unit located at the prosthetic shank adapter to determine gait situation, velocity and phase. To achieve the user-specific system behavior, high-level control selects the desired system behavior parameters from the IPSM based on the detected gait situation whereas low-level control commands the actuator torque.

### 4.1. High-Level Control

The high-level control strategy is developed to assist the user considering gait situation and gait phase. Therefore, gait direction, velocity, and phases are identified to select the desired system behavior depending on gait situation and amputee's preference. The corresponding parameters are transmitted to the low-level control. A gait detection algorithm processes signals from an inertial measurement unit (BNO 055, Robert Bosch GmbH, Gerlingen, Germany) implemented in the structure of the prosthesis. In preliminary investigations with healthy users (Schuy et al., 2015), angular shank velocity in sagittal plane ω_*ML*_ and frontal plane ω_*AP*_ acquired from gyroscopes were sufficient to determine gait direction, detect stance and swing phase and estimate gait velocity.

**Figure 3:**
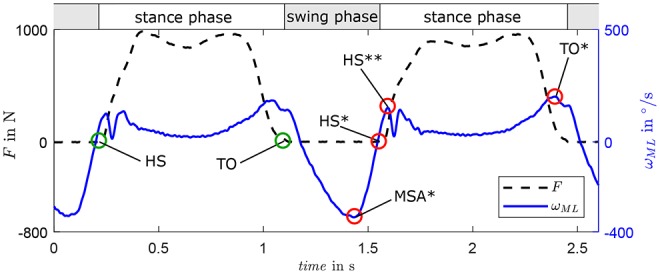
Behavior of ground reaction force *F* in longitudinal axis of the shank and angular shank velocity in the sagittal plane ω_*ML*_ for two exemplary gait cycles with characteristic gait events heel strike (HS) and toe-off (TO) during two steps. Event estimations are marked with * and HS** represents the HS-related force peak considered in some algorithms.

Gait phase estimation is based on the identification of characteristic events of stance and swing phase. Figure 3 displays the vertical force (black dotted) of two steps as reference. Stance phases are characterized by positive force values and separated from swing phases by Heel Strike (HS) and Toe Off (TO), which are both marked by green circles. Characteristic events of ω_*ML*_ (blue solid line) are used to identify the gait phases. HS is estimated considering the zero-crossing HS* of the signal (Behboodi et al., 2015; Schuy, 2016) instead of the peak HS** that is used in many other studies (Aminian et al., 2002; Jasiewicz et al., 2006; Catalfamo et al., 2010; Greene et al., 2010; Lee and Park, 2011; Gouwanda and Gopalai, 2015). The end of stance phase (TO) is estimated by the maximum amplitude TO*.

The minimum amplitude in swing phase MSA* (see Figure 3) is used to estimate gait velocity since both exhibit a quasi-linear correlation as tested on a treadmill with healthy users (Schuy, 2016). This correlation is user-dependent and should be determined individually, e.g., in walking trials during the fitting process.

**Figure 4:**
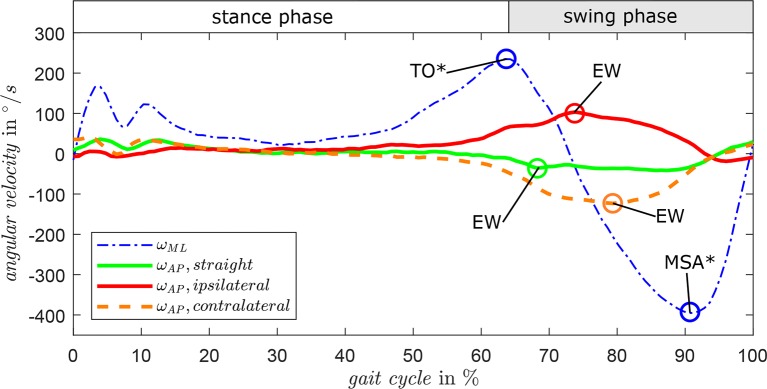
Angular shank velocity in sagittal plane ω_*ML*_ and frontal plane ω_*AP*_ for exemplary gait cycles in different turning directions. By detecting the maximum amplitude (EW) of ω_*AP*_ during TO* and MSA*, the direction of straight, turning right, and turning left can be classified by a fuzzy algorithm.

Gait direction estimation is based on sensor data of ω_*AP*_. Figure 4 presents the characteristic progression of ω_*AP*_ for straight walking (green), ipsilateral turning (red), and contralateral turning (orange) of the step before a direction change. For reference, ω_*ML*_ is displayed with the characteristic events TO* and MSA* of one gait cycle. By detecting the maximum amplitude (EW) between TO* and MSA*, the gait direction can be classified by a hybrid model of a fuzzy system. This fuzzy logic approach enables a user-specific distinction of the basic three directions require user-specific training of the algorithm to achieve satisfying detection rates based on individual smooth thresholds. Basic tests with non-amputees showed individual detection rates between 91.4 and 100 % in a course around obstacles. Functional tests with participants with amputation confirm the reliable applicability of the detection algorithm (Schuy, 2016), which is also underlined by a previous study considering 3, 000 steps of 15 able-bodied personens and 2 persons with amputation on a predefined parcours achieving comparable detection rates distinctly above 90% and similar reliability (Schuy et al., 2015).

Based on the high-level control algorithm information about gait direction, phase, and velocity are gathered. Using these information the parameter of individual optimal torsional stiffness and transversal foot alignment are selected from the IPSM to be used in the low-level control algorithm to adjust the prosthetic shank adapter.

### 4.2. Low-Level Control

**Figure 5:**
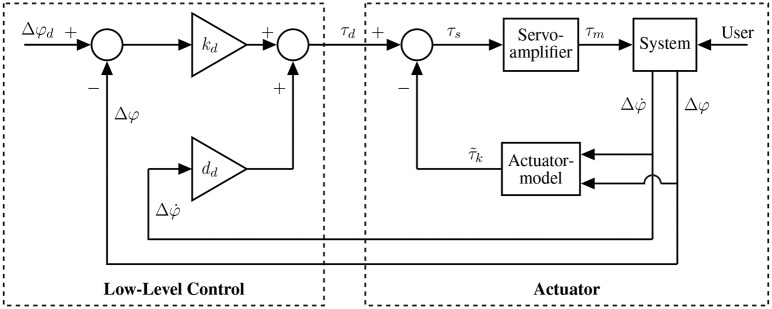
Control scheme for calculating motor torque to set the desired system behavior. The low-level control is realized as a first-order impedance control, directly incorporating the user-preferred torsional stiffness as a control parameter.

The low-level control algorithm transforms the desired system behavior into a command signal for the actuator. Therefore, the control scheme depicted in Figure 5 is implemented with the objective to generate a spring-like reaction to external loads with the desired stiffness *k*_*d*_, which is equivalent to the user-specific optimal torsional stiffness as determined by the high-level control algorithm. The corresponding desired torque τ_*d*_ is determined from desired foot alignment Δφ_*d*_ and actual foot alignment Δφ by

(1) $$\begin{array}{l} \tau_{d}=k_{d}\left(\Delta \varphi_{d}-\Delta \varphi \right)+d_{d}\Delta \dot{\varphi }. \end{array}$$

Additional viscous damping *d*_*d*_ provides smooth trajectories. This control scheme directly creates the user-specific, optimal stiffness in the stance phase. To control the foot alignment in swing, without external loads at the foot, τ_*d*_ is set to achieve an equilibrium with the torque of the spring at Δφ_*d*_.

### 4.3. Optimization

To achieve a high power-to-weight ratio and an energy efficient human-machine interaction, optimization of topology and parameters of the actuator is performed based on the test-cycle presented in section 3. Therefore, direct drive (DD), serial-elastic actuator (SEA), and parallel-elastic actuator (PEA) are considered as basic topologies (Van Ham et al., 2009) in this paper. While SEAs mimic the conceptual structure of muscle-tendon complexes (muscle = actuator, tendon = spring), a PEA can implement a simplistic imitation of the musculoskeletal structure of the shank (tibia & fibula = torsion spring, muscles = actuator). Table 4 presents the dynamics models and equations of DD (left), SEA (middle), and PEA (right). The expressions τ_*a*_ and φ_*a*_ describe the actuator torque and angle. Inertia of motor and gearbox are given by θ_*m*_ and θ_*g*_. The parallel and series stiffness are denoted by *k*_*p*_ and *k*_*s*_, respectively.

**Table 4:** Models and equations of motion and motor torque for a direct drive, serial-elastic actuator and parallel-elastic actuation systems, employing a motor with transmission (Schuy, 2016).

**DD**	**SEA**	**PEA**
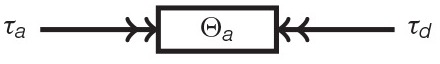	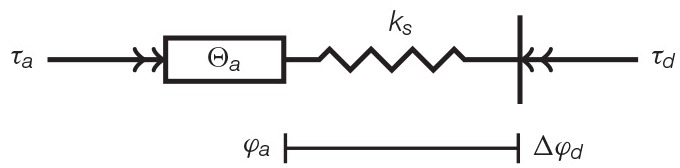	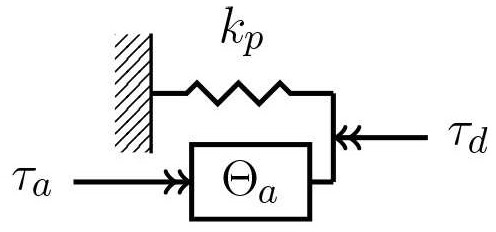
φ_*a*_ = Δφ	$\varphi_{a}=\Delta \varphi +\frac{\tau_{d}}{k_{s}}$	φ_*a*_ = Δφ
${\dot{\varphi }}_{m}={\dot{\varphi }}_{a}i_{g}=\Delta \dot{\varphi }i_{g}$	${\dot{\varphi }}_{m}={\dot{\varphi }}_{a}i_{g}=\left(\Delta \dot{\varphi }+\frac{{\dot{\tau }}_{l}}{k_{s}}\right)i_{g}$	${\dot{\varphi }}_{m}={\dot{\varphi }}_{a}i_{g}=\Delta \dot{\varphi }i_{g}$
$\tau_{m}=\left(\theta_{m}+\frac{\theta_{g}}{i_{g}^{2}}\right)\Delta \ddot{\varphi }i_{g}+$	$\tau_{m}=\left(\theta_{m}+\frac{\theta_{g}}{i_{g}^{2}}\right)\left(\Delta \ddot{\varphi }+\frac{{\ddot{\tau }}_{l}}{k_{s}}\right)i_{g}+$	$\tau_{m}=\left(\theta_{m}+\frac{\theta_{g}}{i_{g}^{2}}\right)\Delta \ddot{\varphi }i_{g}+$
$+\frac{C}{i_{g}}\tau_{d}$	$+\frac{C}{i_{g}}\tau_{d}$	$+\frac{C}{i_{g}}\left(\tau_{d}+k_{p}\Delta \varphi \right)$

The transmission with gear ratio *i*_*g*_ leads to the motor angle φ_*m*_. In addition to the gear ratio, the efficiency of the gear unit η is considered and assumed to be 0.8, impacting the torque τ_*m*_ on the motor shaft (Verstraten et al., 2016). Depending on the direction of the power, the efficiency is written as:

(2) $$\begin{array}{l} C=\{\begin{array}{cc} \frac{1}{\eta } & \text{load driven by motor}\\ \eta & \text{motor driven by load} \end{array} \end{array}$$

Based on the equations of motion given in Table 4, the current *I* of a direct current motor can be determined from:

(3) $$\begin{array}{l} I=\frac{\tau_{m}+d_{m}{\dot{\varphi }}_{m}}{k_{m}} \end{array}$$

Where *k*_*m*_ is the torque constant and *d*_*m*_ the viscous damping coefficient of the motor. The latter can be estimated from the no-load speed ${\dot{\varphi }}_{nl}$ and the no-load current *I*_*nl*_ according to:

(4) $$\begin{array}{l} d_{m}=\frac{k_{m}I_{nl}}{{\dot{\varphi }}_{nl}} \end{array}$$

This allows the determination of the motor voltage considering the speed constant *c*_*n*_:

(5) $$\begin{array}{l} U=L\dot{I}+RI+\frac{{\dot{\varphi }}_{m}}{c_{n}} \end{array}$$

In the following, the electrical power *P* = *UI*, is used to determine the required peak power *P*_*p*_

(6) $$\begin{array}{l} P_{p}=\text{max}\left(\left|P\right|\right) \end{array}$$

and electrical energy per test-cycle *E*

(7) $$\begin{array}{l} E=\int |P|\text{d}t \end{array}$$

for DD, PEA, and SEA.

To minimize either peak power or consumed energy, brute-force optimizations are conducted by varying *k*_*s*_ or *k*_*p*_ from 0.01 N m/° to 10 N m/° with a step-width of 0.01 N m/°. Spring behavior is assumed to be linear and friction as well as recuperation are neglected, which is sufficient for optimization (Verstraten et al., 2016). In order to determine the electrical power, the brushless direct current motor EC40i (50 W, Maxon Motor, Sachseln, Switzerland) and gearbox GP32C (Maxon Motor, Sachseln, Switzerland) with a ratio of 103:1 are preselected.

**Figure 6:**
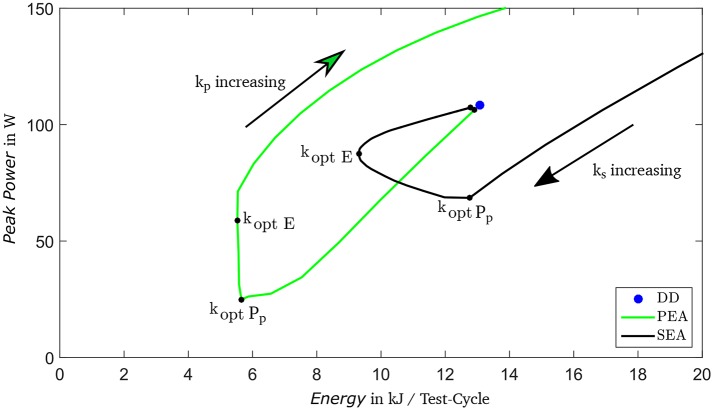
Comparison of Peak Power (PP) and Energy Consumption (E) per test-cycle for varying spring stiffness in three drive train topologies: serial-elastic actuator (SEA), parallel-elastic actuator (PEA), and direct drive (DD).

The comparison of energy consumption (*E*) and peak power (*P*_*p*_) in Figure 6 shows advantages of the highlighted optimization results for the PEA compared to the DD and the SEA. The optimal stiffness for the PEA in terms of energy consumption *k*_*opt E*_ is 1.06 N m/° and in terms of peak power *k*_*optP*_*p*__ = 0.76 N m/°. A PEA implementing *k*_*optP*_*p*__ would exhibit distinctly lower peak power and similar energy consumption compared to one with *k*_*opt E*_. Hence, *k*_*optP*_*p*__ is selected for implementation in the hardware system.

In order to verify the preselected motor and gearbox, the required torque in dependency of angular velocity and desired system stiffness for distinct gait situations is analyzed. The selected drive train requires a maximal peak torque of 128% of nominal torque (for circling, ipsilateral) and is therefore advantageous compared to the SEA with 355% of nominal torque and DD with 359%, respectively. Further, in case of motor break down the PEA concept provide a safe state with characteristics of a passive device. For more detailed information (see Schuy, 2016).

To consider the effect of the parallel spring in the control strategy, an actuator model is included as depicted in Figure 5. Thus, the required amplifier input is simplified to:

(8) $$\begin{array}{l} \tau_{s}=\tau_{d}-{\tilde{\tau }}_{k}. \end{array}$$

To avoid additional weight and complexity of a torque sensor, ${\tilde{\tau }}_{k}$ is a neural-network-based estimation of the elastic torque in the parallel spring. The neural network facilitates considering motor inertia as well as nonlinear effects such as friction and hysteresis as identified in bench-testing. Ten hidden neurons were fed with mid swing and heel strike amplitude date and trained using a Levenberg-Marquardt algorithm (Schuy et al., 2015). Training and evaluation was performed with different data subsets from the preliminary study by Schuy et al. (2015) considering different gait situations and speeds. The current control of the motor controller EPOS 24/5 (Maxon Motor AG, Sachseln, Switzerland) is tuned to generate the motor torque τ_*m*_ based on the input τ_*s*_ while considering the gear ratio.

### 4.4. Implementation

**Figure 7:**
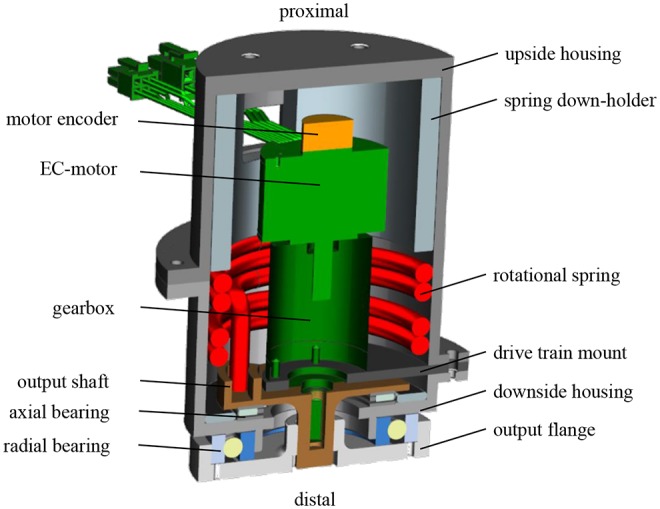
Cross section of the developed prosthetic shank adapter, featuring the electric motor with gearbox and rotational springs in a parallel-elastic configuration (Schuy, 2016).

The developed PEA concept for adjustable torsional stiffness and foot alignment is implemented in the prosthetic shank adapter, as shown in Figure 7. The aluminum housing carries the functional parts and serves as flange to connect it with standardized prosthetic pyramid adapters. On the distal side of the prosthetic shank adapter, a radial ball bearing plus a needle bearing provide the rotational degree of freedom while taking loads. Two rotational springs are used to cover both turning directions, mounted with pretension to avoid backlash, and implement the transverse plane elastic properties of tibia and fibula. The prototype was tested successfully by Chas A. Blatchford & Sons Ltd. (Basingstoke, United Kingdom), according to the structural test by ISO 10328 (A80 P4) and was approved for preliminary user testing.

An encoder at the motor measures the relative angle while acceleration and velocity of the shank are captured by an inertial sensor unit BNO 55 (Robert Bosch GmbH, Germany). It is mounted between the prototype housing and the pyramid adapter on the proximal side. The device exhibits an axial height of 0.127 m, a diamter of 0.1 m, and a weight of 1.39 kg without the backpack containing power supply and control hardware (Schuy, 2016). The motor controller, the main controller myRIO (National Instruments, Austin, U.S.) as well as a lithium polymer battery as power supply are located in a backpack for user tests.

## 5. Evaluation

**Figure 8:**
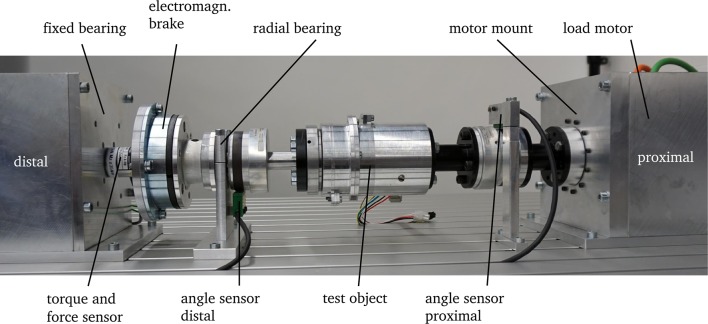
Test rig with integrated adaptive prosthetic shank adapter as the test object. The load motor and electromagnetic brake are used to mimic the motion and load of the shank.

Before fitting the adaptive prosthetic shank adapter to users, the prototype is functionally tested via simulations and bench testing. Therefore, the test rig presented in Figure 8 was developed. It contains a load motor (CHA-17A-80H-M128S Harmonic Drive AG, Limburg a. d. Lahn, Germany) that generates the proximal load, which is applied to the test object analog to rotation load to during gait. The rotational angle is measured proximal and distal (MR210 and MSK210, SIKO GmbH, Buchenbach, Germany) with magnetic incremental sensors. The distal degree of freedom is either locked during stance to emulate contact between foot and ground or can rotate freely in the swing phase. An electromagnetic friction brake (Combinorm-B 08.02.320, KEB GmbH, Barntrup, Germany) is used to generate a sufficiently high locking torque. The distal load is measured by a torque transducer (ALF 310-Z, ALTHEN GmbH, Kelkheim, Germany). Applying motion and load of the test cycle proposed in section 3 allows for an evaluation of the mechanics and control performance under realistic conditions.

**Figure 9:**
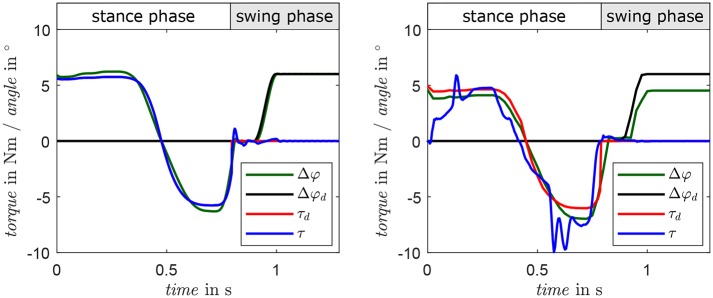
Results of a comparison of a 90°-turn in simulation **(left)** and system behavior at the test bench **(right)**. Measured torque is presented in blue, desired torque in red. Actual and desired motor angles are depicted in green and black, respectively.

A comparison of simulation and bench testing is given in Figure 9. The desired foot alignment is set to 6° for the complete step while the system is subjected to a turning motion taken from the preliminary experiments (Schuy, 2016). In the interval from 0.4 to 0.8 s, the external load has a considerable amplitude and the system shows the desired, spring-like characteristic, as measured torque follows the desired curve. In swing phase, the foot alignment achieves the desired position after approximately 0.2 s and remains constant in preparation for the next step. The feasibility of the control strategy as well as the user-specific stiffness and foot alignment is pointed out by the simulation results that show good position and torque tracking. Yet, deviations occur in the measured torque due to nonlinearities in the drive unit, which are not adequately described by the actuator model utilized in the control strategy. In the swing phase, the angle of the foot Δφ almost reaches the desired alignment Δφ_*d*_. The remaining angular difference occurs due to underestimation of friction in the gear unit. Consequentially, the continuous motor current reaches 5 A, which is the limit of the utilized motor driver. To avoid overheating, the motor current is limited and an equilibrium between motor torque and deflection of the parallel springs is set.

## 6. Basic User Testing

**Figure 10:**
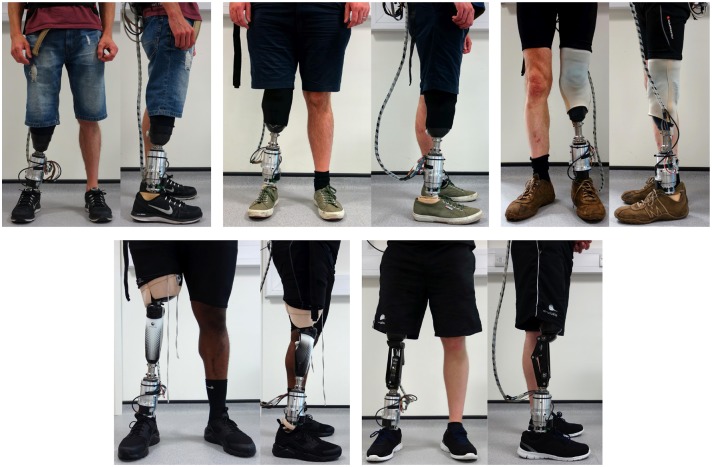
The prosthetic shank adapter is implemented and aligned to the prosthetic structure of three transtibial **(top)** and two transfemoral **(bottom)** amputees for calibration and basic tests (Schuy, 2016).

After extensive bench-testing, the prototype was tested by five participants with lower limb amputation (three transtibial, two transfemoral). The experiments were conducted with a positive vote by the ethics committee of the Technische Universität Darmstadt and in accordance with the Declaration of Helsinki in its current version. For the tests, the participants' everyday prostheses were equipped with the prosthetic shank adapter as shown in Figure 10.

**Figure 11:**
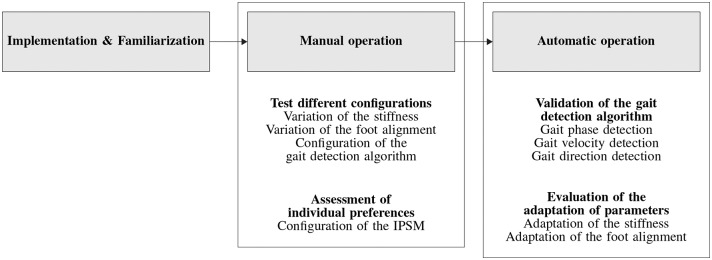
Procedure to configure and evaluate the prosthetic shank adapter throughout the basic tests.

After providing informed consent, participants were asked to perform different tasks at self-selected slow, medium and fast velocity (straight walking, clockwise, and counterclockwise circling with a radius of 1 m as well as walking around a 90° corner in left and right direction) while the prosthetic shank adapter is calibrated according to the procedure presented in Figure 11. Subsequently, participants performed the tasks with deactivated adaptation, to familiarize themselves to the weight and the passive torsional stiffness of the system. Data acquired during this acclimation phase was used to tune and validate the gait detection algorithms. Afterwards, single functions and the behavior of the system are configured by hand in manual mode of the device. This allows to test different gait situations as well as configurations of optimal torsional stiffness and foot alignment to refine the individual preference setting matrix (Schuy, 2016). After manual mode operation and testing, the automatic operation is activated to perform adaptations regarding the gait situation and speed completely independent of a third party.

**Figure 12:**
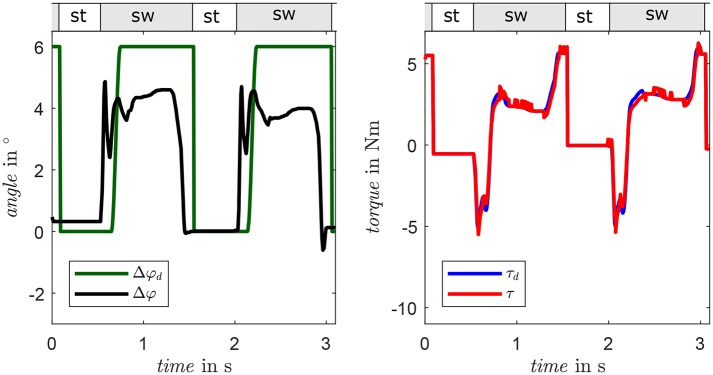
Foot alignment **(left)** and torque **(right)** of the prosthetic shank adapter of one representative participant performing a clockwise circling motion (prosthesis inside). The stance phase and swing phase are labeled with st and sw, respectively.

In the manual operation trials, all participants reported that the weight of the device is not relevant and that they experienced distinct benefits in the tested gait situations using their preferred setups. Figure 12 presents a clockwise circling motion with a radius of 1 m at self-selected medium gait velocity performed by a participant, whereby the prosthesis is inside. Gait phase and gait situation are detected correctly and the desired foot alignment is set to 6° during swing accordingly. While the foot alignment does not reach the maximum desired value as depicted in the left of Figure 12, the delay between desired foot alignment and gait phase is devised intentionally to set the desired torque via the control algorithm. The actual torque follows the desired value very well as depicted in the right of Figure 12, exhibiting the desired spring-like characteristics. The automatic mode was tested with two participants (one with transtibial, one with transfemoral amputation). When using automatic operation, the participants particularly highlighted the turning support when walking freely as well as in different velocities during straight level walking. This subjective feedback substantiates the assisting function of the proposed prosthetic shank adapter with adaptable torsion stiffness and foot alignment. Further, it confirms the functionality of the adapter by gait speed and situation detection by the presented algorithms as well as system design and behavior based on the IPSM.

## 7. Discussion

Torsion adapters can reduce stress at the stump of people with lower limb amputation and enable them to turn faster and with less effort. In contrast to existing devices with passive mechanics, this paper suggests a novel bio-inspired mechatronic device with user-specific human-machine interaction properties. This is achieved by adaptation of the torsional stiffness in stance phase and transversal foot alignment during swing phase for different gait situations and speeds. Bench and user trials outline that the system is capable to mimic the functionality of the human shank and its positive impact on user experience.

A test-cycle is developed by combining statistical information about daily movements with measured load data from the preliminary study. It merges the load and movement data of several single gait situations to model human motions in defined environments to mimic a typical day of persons with lower limb amputation.

For adequate timing and system behavior, a high-level control is realized to define the characteristics of the human-machine interaction. It includes a gait detection algorithm which is based on non-amputees data and adapted to the gait behavior of amputees. By identifying the gait phases, gait velocity, and gait situations, optimal parameters for torsional stiffness and foot alignment are chosen from the individual preference-setting matrix. The low-level control algorithm generates a actuator commands in order to realized the desired system behavior.

Based on the test-cycle, elastic drive-train concepts are examined to identify an optimal mechatronic design. Comparing a serial-elastic drive train and a parallel-elastic drive train to a direct drive shows that the elastic concepts can reduce maximum peak power and energy consumption. The parallel-elastic concept exhibits the lowest peak power and second lowest energy consumption and is thus designed and realized in a proof-of-concept model. This parallel-elastic design is in analogy with the structure of the human shank, i.e., springs resembling bone elasticity and a motor mimicking muscular activity. To control the motor, a low-level control is developed including the non-linear hardware behavior as well as a friction compensation.

The realized hardware and low-level control is verified on a test bench. The high-level control is tested with non-amputees in treadmill tests as well as a course of an eight and free walking. Finally, the overall performance and user experience of the system is validated in trials with five participants with transtibial and transfemoral amputations. They report reduced stress at the residual limb, which coincides with results found in a pilot study (Pew and Klute, 2017a), however, only the torsional stiffness was adjusted in the employed device. In addition, participants reported easier turning and increased stability while walking with different velocities due to the adaptive system behavior. Additional trials with completely autonomous operation of the device underline the proper function of its hardware design and control algorithms as well as the resulting user benefits. In further investigations a higher number of participants should be tested with a miniaturized device in clinical studies. This would enable a validation and elaboration of the individual preference-setting matrix. Additionally, the hardware design should be improved by reducing weight and friction, which would also support improvements of the control and adaptation algorithms. For comparison, commercially available passive rotation adapters exhibit weights between 0.19 and 0.61 kg depending on the application, but certainly lack functionality and adaptivity. A machine-learning approach might be employed to improve the gait detection algorithm. Especially, identifying the end of stance phase and the gait velocity estimation are challenging in slow gait situations and untypical gait patterns. Further, a more detailed differentiation of gait direction, e.g., of slight turnings, might enable a higher number of defined system behavior setups and thus an increased benefit to the user.

## Data Availability Statement

The datasets generated for this study are available on request to the corresponding author.

## Ethics Statement

The studies involving human participants were reviewed and approved by Ethics committee of TU Darmstadt, Germany. The patients/participants provided their written informed consent to participate in this study.

## Author Contributions

JS designed the prosthetic device as well as coordinated and conducted the study. PB supported the design process, especially regarding actuator and controls. PB and SR supervised device development and implementation as well as study preparation. NS, GH, and SZ supported and supervised the mechanical design and testing of the device as well as the trials with human participants. All authors contributed to writing the manuscript, reviewed, and confirmed the submitted version.

## Conflict of Interest

JS is employed by Continental Teves AG & Co. oHG, Germany. NS is employed by seleon gmbh, Germany. GH and SZ are employed by Chas A Blatchford & Sons Ltd, Basingstoke, Hampshire, United Kingdom. The remaining authors declare that the research was conducted in the absence of any commercial or financial relationships that could be construed as a potential conflict of interest.

## References

[B1] Aminian K. Najafi B. Büla C. Leyvraz P.-F. Robert P. (2002). Spatio-temporal parameters of gait measured by an ambulatory system using miniature gyroscopes. J. Biomech. 35, 689–699. 10.1016/S0021-9290(02)00008-8 11955509

[B2] Beckerle P. Christ O. Schürmann T. Vogt J. von Stryk O. Rinderknecht S. (2017). A human-machine-centered design method for (powered) lower limb prosthetics. Robot. Auton. Syst. 95, 1–12. 10.1016/j.robot.2017.05.004

[B3] Behboodi A. Wright H. Zahradka N. Lee S. (2015). Seven phases of gait detected in real-time using shank attached gyroscopes, in Engineering in Medicine and Biology Society (EMBC), 2015 37th Annual International Conference of the IEEE (Milan), 5529–5532. 10.1109/EMBC.2015.7319644 26737544

[B4] Catalfamo P. Ghoussayni S. Ewins D. (2010). Gait event detection on level ground and incline walking using a rate gyroscope. Sensors 10, 5683–5702. 10.3390/s100605683 22219682 PMC3247727

[B5] Flick K. Orendurff M. Berge J. Segal A. Klute G. (2005). Comparison of human turning gait with the mechanical performance of lower limb prosthetic transverse rotation adapters. Prosthet. Orthot. Int. 29, 73–81. 10.1080/03093640500088120 16180379

[B6] Gard S. Konz R. (2003). The effect of a shock-absorbing pylon on the gait of persons with unilateral transtibial amputation. J. Rehabil. Res. Dev. 40, 109–124. 10.1682/JRRD.2003.03.0109 15077637

[B7] Glaister B. (2012). Controllable Transverse Rotation Adaptor. US Patent 8,598,815. Available online at: https://patents.google.com/patent/US8598815

[B8] Glaister B. Orendurff M. Schoen J. Klute G. (2007). Rotating horizontal ground reaction forces to the body path of progression. J. Biomech. 40, 3527–3532. 10.1016/j.jbiomech.2007.05.014 17597134

[B9] Glaister B. Schoen J. Orendurff M. Klute G. (2009). A mechanical model of the human ankle in the transverse plane during straight walking: implications for prosthetic design. J. Biomech. Eng. 131:034501. 10.1115/1.3005153 19154072

[B10] Glaister C. Bernatz G. Klute G. Orendurff M. (2007). Video task analysis of turning during activities of daily living. Gait Posture 25, 289–294. 10.1016/j.gaitpost.2006.04.003 16730441

[B11] Gouwanda D. Gopalai A. (2015). A robust real-time gait event detection using wireless gyroscope and its application on normal and altered gaits. Med. Eng. Phys. 37, 219–225. 10.1016/j.medengphy.2014.12.004 25619613

[B12] Greene B. McGrath D. O'Neill R. O'Donovan K. Burns A. Caulfield B. (2010). An adaptive gyroscope-based algorithm for temporal gait analysis. Med. Biol. Eng. Comput. 48, 1251–1260. 10.1007/s11517-010-0692-0 21042951

[B13] Halsne E. Waddingham M. Hafner B. (2013). Long-term activity in and among persons with transfemoral amputation. J. Rehabil. Res. Dev. 50, 515–530. 10.1682/JRRD.2012.04.0066 23934872

[B14] Hansen A. H. Childress D. S. Miff S. C. Gard S. A. Mesplay K. P. (2004). The human ankle during walking: implications for design of biomimetic ankle prostheses. J. Biomech. 37, 1467–1474. 10.1016/j.jbiomech.2004.01.017 15336920

[B15] Heitzmann D. Pieschel K. Alimusaj M. Block J. Putz C. Wolf S. (2015). Functional effects of a prosthetic torsion adapter in trans-tibial amputees during unplanned spin and step turns. Prosthet. Orthot. Int. 40, 558–565. 10.1177/0309364615592698 26195621

[B16] Hordacre B. Barr C. Crotty M. (2014). Use of an activity monitor and gps device to assess community activity and participation in transtibial amputees. Sensors 14, 5845–5859. 10.3390/s140405845 24670721 PMC4029655

[B17] Jasiewicz J. Allum J. Middleton J. Barriskill A. Condie P. Purcell B.. (2006). Gait event detection using linear accelerometers or angular velocity transducers in able-bodied and spinal-cord injured individuals. Gait Posture 24, 502–509. 10.1016/j.gaitpost.2005.12.017 16500102

[B18] Klute G. Berge J. Orendurff M. Williams R. Czerniecki J. (2006). Prosthetic intervention effects on activity of lower-extremity amputees. Arch. Phys. Med. Rehabil. 87, 717–722. 10.1016/j.apmr.2006.02.007 16635636

[B19] Lamoureux L. Radcliffe C. (1977). Functional analysis of the UC-BL shank axial rotation device. Prosthet. Orthot. Int. 1, 114–118. 10.3109/03093647709164619 615294

[B20] Lee J. Park E. (2011). Quasi real-time gait event detection using shank-attached gyroscopes. Med. Biol. Eng. Comput. 49, 707–712. 10.1007/s11517-011-0736-0 21267666

[B21] Lelas J. Merriman G. Riley P. Kerrigan D. (2003). Predicting peak kinematic and kinetic parameters from gait speed. Gait Posture 17, 106–112. 10.1016/S0966-6362(02)00060-7 12633769

[B22] Nietert M. Englisch N. Kreil P. Alba-Lopez G. (1997). International Study on the Acquistion of Loads in Hip Disarticulation Prostheses. Technical report, Department of Hospital and Biomedical Engineering, Enviromental and Biotechnology Fachhochschule, Technical College.

[B23] Nietert M. Englisch N. Kreil P. Alba-Lopez G. (1998). Loads in hip disarticulation prostheses during normal daily use. Prosthet. Orthot. Int. 22, 199–215. 10.3109/03093649809164485 9881608

[B24] Oehler S. (2015). Mobilitätsuntersuchungen und Belastungsmessungen an Oberschenkelamputierten, Vol. 3 (Walter de Gruyter GmbH & Co KG). 10.1515/9783110267860

[B25] Office of Mobile Sources and Offif of Air & Radiation (1993). Federal Test Procedure Review Project: Preliminary Technical Report. Technical Report, U.S Enviromental Protection Agency.

[B26] Olson N. Klute G. (2015). Design of a transtibial prosthesis with active transverse plane control. J. Med. Dev. 9:045002 10.1115/1.4031072

[B27] Orendurff M. (2012). Dynamic foot and ankle characteristics in functionally relevant gait performance in those with and without a pathology (Ph.D. thesis). University of Roehampton, London, United Kingdom.

[B28] Pew C. (2014). Design and testing of a variable stiffness transverse plane adaptor for use in a lower limb prosthesis (Ph.D. thesis). University of Washington, Seattle, WA, United States.

[B29] Pew C. Klute G. (2015). Design of lower limb prosthesis transverse plane adaptor with variable stiffness. J. Med. Devices 9:035001 10.1115/1.4030505

[B30] Pew C. Klute G. K. (2017a). Pilot testing of a variable stiffness transverse plane adapter for lower limb amputees. Gait Posture 51, 104–108. 10.1016/j.gaitpost.2016.10.003 27744248

[B31] Pew C. Klute G. K. (2017b). Second generation prototype of a variable stiffness transverse plane adapter for a lower limb prosthesis. Med. Eng. Phys. 49, 22–27. 10.1016/j.medengphy.2017.07.002 28807513

[B32] Price M. A. Beckerle P. Sup F. C. (2019). Design optimization in lower limb prostheses: a review. IEEE Trans. Neural Syst. Rehabil. Eng. 27, 1574–1588. 10.1109/TNSRE.2019.2927094 31283485

[B33] Ross J. Luff R. Ledger M. (2003). Study of Telescopic Pylon on Lower Limb Amputees. Ortho. Tech. Q. 111, 4–1.

[B34] Schuy J. (2016). Variable Torsionssteifigkeit in Unterschenkelprothesen zur aktiven Unterstützung in dynamischen Gangsituationen (Ph.D. thesis). TU Darmstadt, Darmstadt, Germany.

[B35] Schuy J. Burkl A. Beckerle P. Rinderknecht S. (2014). A new device to measure load and motion in lower limb prosthesis - tested on different prosthetic feet, in 2014 IEEE International Conference on Robotics and Biomimetics (ROBIO) (Bali), 187–192. 10.1109/ROBIO.2014.7090328

[B36] Schuy J. Mielke T. Steinhausen M. Beckerle P. Rinderknecht S. (2015). Design & evaluation of a sensor minimal gait phase and situation detection algorithm of human walking, in 2015 IEEE-RAS 15th International Conference on Humanoid Robots (Humanoids) (Seoul), 20–25. 10.1109/HUMANOIDS.2015.7363517

[B37] Schuy J. Rinderknecht S. (2014). Integrated measurement system for amputee gait analysis: a pilot study, in Healthcare Innovation Conference (HIC), 2014 IEEE (Seattle, WA), 91–94. 10.1109/HIC.2014.7038882

[B38] Sedgman R. Goldie P. Iansek R. (1994). Development of a measure of turning during walking, in Advancing rehabilitation: Proceedings of the Advancing Rehabilitation Conference (Melbourne, VIC: La Trobe University), 26–31.

[B39] Segal A. Kracht R. Klute G. (2014). Does a torsion adapter improve functional mobility, pain, and fatigue in patients with transtibial amputation? Clin. Orthop. Relat. Res. 472, 3085–3092. 10.1007/s11999-014-3607-9 24733445 PMC4160517

[B40] Segal A. Orendurff M. Czerniecki J. Schoen J. Klute G. (2011). Comparison of transtibial amputee and non-amputee biomechanics during a common turning task. Gait Posture 33, 41–47. 10.1016/j.gaitpost.2010.09.021 20974535

[B41] Segal A. Orendurff M. Czerniecki J. Shofer J. Klute G. (2009). Transtibial amputee joint rotation moments during straight-line walking and a common turning task with and without a torsion adapter. J. Rehabil. Res. Dev. 46, 375–383. 10.1682/JRRD.2008.06.0070 19675989

[B42] Segal A. Orendurff M. Czerniecki J. Shofer J. Klute G. (2010). Local dynamic stability of amputees wearing a torsion adapter compared to a rigid adapter during straight-line and turning gait. J. Biomech. 43, 2798–2803. 10.1016/j.jbiomech.2010.05.038 20719315

[B43] Stauf C. (2000). Untersuchung der prothesen-rotationsstossdampfer OSI und USI im rahmen einer biomechanikstudie. Ortho. Tech. 267–270.

[B44] Stepien J. Cavenett S. Taylor L. Crotty M. (2007). Activity levels among lower-limb amputees: self-report versus step activity monitor. Arch. Phys. Med. Rehabil. 88, 896–900. 10.1016/j.apmr.2007.03.016 17601471

[B45] Strike S. Taylor M. (2009). The temporal-spatial and ground reaction impulses of turning gait: is turning symmetrical? Gait Posture 29, 597–602. 10.1016/j.gaitpost.2008.12.015 19195890

[B46] Stuhlenmiller F. Schuy J. Beckerle P. Rinderknecht S. (2017). A user-specific human-machine interaction strategy for a prosthetic shank adapter. Curr. Direct. Biomed. Eng. 3, 493–496. 10.1515/cdbme-2017-0103

[B47] Su P.-F. Gard S. Lipschutz R. Kuiken T. (2010). The effects of increased prosthetic ankle motions on the gait of persons with bilateral transtibial amputations. Am. J. Phys. Med. Rehabil. Assoc. Acad. Physiatr. 89:34. 10.1097/PHM.0b013e3181c55ad4 20026945 PMC2805409

[B48] Taylor M. (2006). A three-dimensional biomechanical analysis of turning gait in both able-bodied and trans-tibial amputee populations (Ph.D. thesis). School of Human and Life Sciences, Roehampton University, University of Surrey, Guildford, England.

[B49] Taylor M. Dabnichki P. Strike S. (2005). A three-dimensional biomechanical comparison between turning strategies during the stance phase of walking. Hum. Mov. Sci. 24, 558–573. 10.1016/j.humov.2005.07.005 16129503

[B50] Tudor-Locke C. Myers A. (2001). Methodological considerations for researchers and practitioners using pedometers to measure physical (ambulatory) activity. Res. Q. Exerc. Sport 72, 1–12. 10.1080/02701367.2001.10608926 11253314

[B51] Twiste M. Rithalia S. (2003). Transverse rotation and longitudinal translation during prosthetic gait-a literature review. J. Rehabil. Res. Dev. 40, 9–18. 10.1682/JRRD.2003.01.0009 15150716

[B52] Van der Linden M. Twiste N. Rithalia S. (2002). The biomechanical effects of the inclusion of a torque absorber on trans-femoral amputee gait, a pilot study. Prosthet. Orthot. Int. 26, 35–43. 10.1080/03093640208726619 12043924

[B53] Van Ham R. Sugar T. Vanderborght B. Hollander K. Lefeber D. (2009). Compliant actuator designs review of actuators with passive adjustable compliance/controllable stiffness for robotic applications. IEEE Robot. Autom. Mag. 16, 81–94. 10.1109/MRA.2009.933629

[B54] Verstraten T. Beckerle P. Dumasl Furnemont R. Mathijssen G. Vanderborght B. (2016). Series and parallel elastic actuation: Impact of natural dynamics on power and energy consumption. Mech. Mach. Theor. 102, 232–246. 10.1016/j.mechmachtheory.2016.04.004

[B55] Windrich M. Grimmer M. Christ O. Rinderknecht S. Beckerle P. (2016). Active lower limb prosthetics: a systematic review of design issues and solutions. Biomed. Eng. Online 15:140. 10.1186/s12938-016-0284-9 28105948 PMC5249019

[B56] Ziegler-Graham K. MacKenzie E. Ephraim P. Travison T. Brookmeyer R. (2008). Estimating the prevalence of limb loss in the United States: 2005 to 2050. Arch. Phys. Med. Rehabil. 89, 422–429. 10.1016/j.apmr.2007.11.005 18295618

